# Subclinical left ventricular dysfunction assessed by global longitudinal strain correlates with mild cognitive impairment in hypertensive patients

**DOI:** 10.1038/s41440-025-02182-3

**Published:** 2025-03-17

**Authors:** Germano Junior Ferruzzi, Alfonso Campanile, Valeria Visco, Francesco Loria, Pasquale Mone, Daniele Masarone, Giuseppe Dattilo, Graziella Agnelli, Alice Moncada, Luigi Falco, Costantino Mancusi, Ilaria Fucile, Pietro Mazzeo, Eugenio Stabile, Rodolfo Citro, William Molloy, Amelia Ravera, Maddalena Illario, Cristina Gatto, Albino Carrizzo, Gaetano Santulli, Guido Iaccarino, Carmine Vecchione, Michele Ciccarelli

**Affiliations:** 1 Department of Medicine, Surgery and Dentistry, University of Salerno, Baronissi, Salerno, Italy; 2 Cardiovascular and Thoracic Department, University Hospital “San Giovanni di Dio e Ruggi d’Aragona”, Salerno, Italy; 3 Department of Medicine and Health Sciences “Vincenzo Tiberio”, University of Molise, Campobasso, Italy; 4 Albert Einstein College of Medicine, New York, NY USA; 5 Casa di Cura Montevergine, GVM Care and Research, Mercogliano, Italy; 6 Heart Failure Unit, AORN Colli, Naples, Italy; 7 Department of Biomedical And Dental Sciences and Morphofunctional Imaging, Section of Cardiology, University of Messina, Messina, Italy; 8 Department of Advanced Biomedical Sciences, Federico II University of Naples, 80138 Naples, Italy; 9 Division of Cardiology, Cardiovascular Department, Azienda Ospedaliera Regionale “San Carlo”, Potenza, Italy; 10 Centre for Gerontology and Rehabilitation, University College Cork, St Finbarr’s Hospital, Cork City, Ireland; 11 Department of Geriatric Medicine, Mercy University Hospital, Cork City, Ireland; 12 Public Health Department, University Federico II of Naples, Naples, Italy; 13 Vascular Pathophysiology Unit, IRCCS Neuromed, Pozzilli, Italy; 14 International Translational Research and Medical Education (ITME) Consortium, Department of Advanced Biomedical Sciences, “Federico II” University, Naples, Italy; 15 Department of Clinical Medicine and Surgery, “Federico II” University, Naples, Italy

**Keywords:** Global longitudinal strain, Vascular risk factors, Cognitive decline, Brain, Propensity weighting analysis

## Abstract

Prevention of dementia represents a public health priority. Hypertension is a risk factor for mild cognitive impairment (MCI), a precursor to progressive dementia. A great effort is underway to develop accurate and sensitive tools to detect the MCI condition in hypertensive patients. To investigate the potential association of subclinical left ventricular dysfunction expressed by the global longitudinal strain (GLS) with the MCI, defined by the Italian version of the quick mild cognitive impairment (Qmci-I). This multi-centric study included 180 consecutive hypertensive patients without medical diseases and/or drugs with known significant effects on cognition but with a not negligible comorbidity burden to avoid a possible “hyper-normality bias”. The study cohort was classified into two main groups concerning the median value of the GLS. A weighted logistic regression model was employed after an inverse probability of treatment weighting (IPTW) analysis to characterize a potential association between GLS and MCI. Almost 41,1% of the whole study population was female. The mean age was 65,6 ± 7,2. 39 patients (21,7%) showed MCI. After IPTW, the GLS was significantly associated with the study endpoint (OR, 1,22; 95% CI: 1,07–1,39, *P* = 0.003). Our results highlight that the GLS is a potential predictor of MCI and, therefore, a valuable tool for establishing preventive strategies to arrest the progression toward a cognitive decline in hypertensive patients.

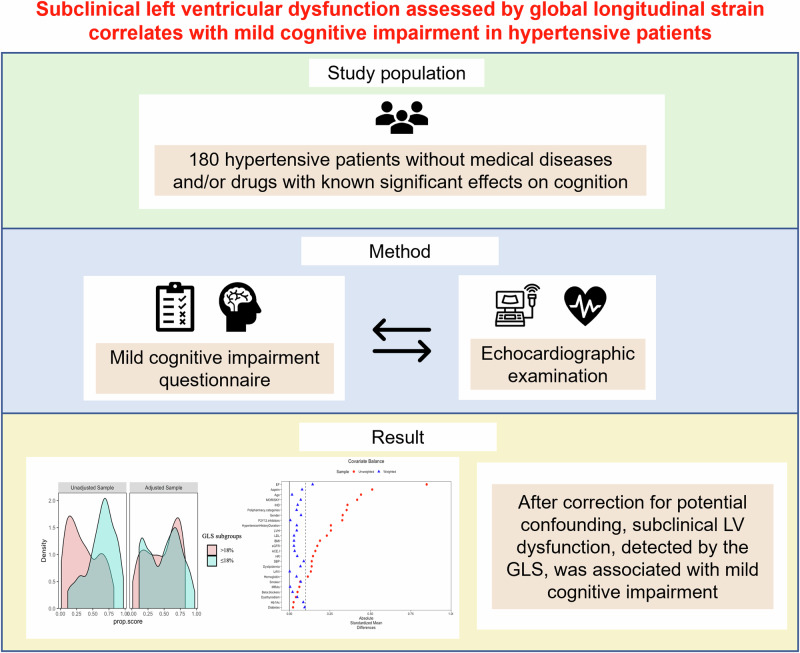

## Introduction

It has been estimated that almost 78 million people by 2030 and 139 million by 2050 will be affected by dementia, with substantial social and economic consequences [1]. Therefore, dementia prevention represents a public health priority [2, 3]. One of the most attainable targets for improving cognitive health among older adults is the tight control of arterial hypertension [4–6]. Indeed, several studies have observed a significant association between midlife hypertension and risk of mild cognitive impairment-MCI (a precursor to progressive dementia) [7]. Recently, the guidelines on hypertension published by the European Societies of Hypertension and Cardiology acknowledge cognitive function (and its decline) as hypertension-mediated organ damage [8]. Specifically, persistently high systemic blood pressure can cause direct modifications of the brain structure or indirectly affect brain function by leading to other syndromes, such as kidney and heart failure [8, 9]. Hypertensive patients are routinely screened for kidney and cardiac dysfunction [8, 10], and, in this specific population, new tools have been developed to detect MCI, which is an early-stage cognitive performance loss, in individuals who still retain the ability to perform most activities of daily living, independently [11]. Given the importance of the early detection of dementia to identify modifiable risk factors before the onset of functional impairment [12], a great effort has been made to develop accurate, reliable, and sensitive tools to improve the detection of MCI [13]. Even if the diagnosis of MCI is mainly clinical, different studies have also investigated the potential predictive role of both neurologic and cardiac imaging parameters in detecting MCI in hypertensive patients [14, 15]. In terms of cardiac function, a promising parameter, in the context of hypertensive cohorts, is represented by the global longitudinal strain (GLS) [16], which appeared to overcome the information provided by LV hypertrophy (LVH), allowing the identification of a subclinical target organ damage [10]. We aimed, therefore, to investigate the potential association of subclinical LV dysfunction quantified by GLS with MCI, defined by the Italian version of quick mild cognitive impairment (Qmci-I), recently developed in Italy in adults over 50 years of age [17].

## Methods

### Study design and participants

We conducted a multi-center observational study in a hypertensive population identified from Cardiology Clinics of 5 Italian University Hospitals, during the year 2023. Inclusion criteria were subjects between 50 and 80 years, with preserved ejection fraction (EF ≥ 50%), and at least 5 years of school education. Exclusion criteria were: the presence of significant neurological and/or psychiatric disorders (including epilepsy, Parkinson’s disease, stroke, psychosis, bipolar disorder, and major depressive disorder); significant general medical diseases interfering with cognition (e.g., atrial fibrillation, significant carotid artery disease with stenosis > 50%); history of alcohol or substance abuse; use of medications with known significant effects on cognition (e.g., anti-psychotics), history of heart failure, and a poor echocardiographic window which strongly limited us to perform the required strain analysis. To avoid a “hyper-normality bias,” disorders usually observed in hypertensive older adults, such as diabetes, hyperlipidemia, ischemic heart disease, and chronic kidney disease, were not considered as exclusion criteria.

Complete demographic, clinical characteristics, laboratory analyses, and echocardiographic data were collected.

All participants provided written informed consent, and the Institutional Research Ethics Committee approved the protocol using the Declaration of Helsinki principles and national regulations.

### Clinical profiling

Hypertension was defined as systolic blood pressure ≥140 mm Hg or diastolic blood pressure ≥90 mmHg or the participant’s self-reported history of hypertension or use of antihypertensive medication, according to the guidelines [8]. Diabetes mellitus was defined as fasting blood glucose ≥126 mg/dL or the participant’s self-reported history of diabetes mellitus or using medications to treat diabetes mellitus. Hypercholesterolemia was defined as total serum cholesterol >240 mg/dL, a patient’s self-report of hypercholesterolemia, or lipid-lowering treatment. Cigarette smoking was recorded during the interview or in the past. Coronary artery disease was defined as a history of myocardial infarction, coronary artery bypass grafting, percutaneous coronary intervention, typical angina, or anti-ischemic medications. Chronic kidney disease was defined as an estimated glomerular filtration rate < 60 mL/min per 1.73 m^2^. The estimated glomerular filtration rate was calculated using the last serum creatinine value available at the time of enrollment or the first value post-enrollment (whichever was closer) with the MDRD study (Modification of Diet in Renal Disease) equation [18].

Self-reporting to assess chronic conditions has been validated as a reliable and cost-effective approach in large-scale population studies [19]. The main medical treatment was reported, and polypharmacy categories were created according to the number of medications used (from 1 to 3, from 4 to 6 and more than 6).

### Cognitive function assessment

Participants completed the Italian version of the quick mild cognitive impairment (Qmci-I) screen. The Qmci-I is composed of six subtests, i.e., orientation, clock drawing, verbal fluency, and three tests of memory (five-word immediate and delayed recall and logical memory-immediate verbal recall of a short story) with scores from 0 (indicating severe impairment) to 100 (indicating higher levels of normal cognition) [13]. It has been showed that the Qmci is a useful test for MCI detection in clinical practice, because of higher sensitivity, in comparison to the standardized Mini-Mental State Examination (MMSE), in differentiating MCI from normal cognition [13].

Every single domain of the Qmci-I was corrected for age and education, and an MCI condition was defined by a total Qmci-I corrected for age and education <49.4 according to the recent literature [17].

Finally, to define compliance with medical treatment, we applied the Morisky medication adherence scale [20].

### Echocardiographic measurements

Echocardiographic examinations were performed with a 3.5 MHz monoplane ultrasound probe of Vivid E-9 (GE-Vingmed Ultrasound, Horten, Norway), according to international guidelines [21]. All parameters were analysed offline by expert operators, blinded to clinical data. The left ventricular ejection fraction (LVEF) was calculated by the Simpson biplane method according to the following formula: LVEF = [left ventricular end-diastolic volume (LVEDV)-left ventricular end-systolic volume (LVESV)]/LVEDV * 100 as a mean of two measures in four and two apical chambers. The mitral E and A velocities, E/A ratio, tissue Doppler analysis of mitral annular E’ velocity, and mitral E/e’ ratio were measured. The presence of a diastolic dysfunction was made according to a specific consensus document [22].

LV hypertrophy was defined, according to the international guidelines [19], as a LV mass index of 125 g/m2 or more in men, and 110 g/m2 or more in women.

LV strain analysis was performed with 2D strain software EchoPAC (GE Healthcare) using high frame rate acquisitions ( > 40 frames per second) of the apical four-chamber, two-chamber, and long-axis view for the LV global longitudinal strain (GLS), as outlined in a specific consensus document [23]. As GLS is a negative value, we used the absolute value of GLS for easier interpretation. According to the GLS median value, our cohort was categorized as having a GLS ≤ 18% or GLS > 18%.

### Statistical analysis

Continuous variables were expressed as mean, standard deviation (SD), or median with interquartile range, depending on the normality of distribution (verified via the Kolmogorov–Smirnov test). Categorical data were expressed as percentages. Comparisons between the two GLS groups were performed using the Student t- and the Mann-Whitney U-test for normally and non-normally distributed continuous variables. Categorical variables were compared using the χ2- or the Fisher exact test when appropriate. The primary study outcome was the prevalence of mild cognitive impairment, defined by a total Qmci-I corrected for age and education <49.4, in the whole study cohort and the GLS subgroups. We first conducted exploratory analysis by testing bivariate unadjusted associations between GLS and LVEF with the total Qmci-I, using Spearman correlations, then we employed an inverse probability of treatment weighting (IPTW) approach to account for the effects of confounding on outcome between the two GLS subgroups (average effect weights).

Inverse probability of treatment weighting is a propensity score method that uses weights based on the propensity score to create a synthetic sample in which the distribution of measured baseline covariates is independent of the GLS median value [24]. In our study, the propensity score was constructed using a logistic regression model that estimated the probability of having a GLS ≤ 18% conditional on the covariates shown in Table 1.

**Table 1:** Clinical characteristics of the whole study cohort, and according to GLS ≤ and > 18%

Variables	Whole study population (*N* = 180)	GLS > 18% (*N* = 92)	GLS ≤ 18% (*N* = 88)	*P* value	SMD before IPTW	SMD after IPTW
***Demographic and clinical data***						
Age	65.6 ± 7.2	64.1 ± 6.9	67.2 ± 7.3	0.003	0.442	0.016
Female gender, N (%)	74 (41.1)	45 (48.9)	29 (33)	0.03	0.329	0.071
Body mass index (BMI)	26,6 (24.4–28.7)	26.7 (24.3–28.7)	26.6 (24.5–28.7)	0.48	0.189	0.028
Systolic blood pressure (mmHg)	130 (120–135)	127.5 (120–140)	130 (120–135)	0.51	0.138	0.088
Heart rate (beats/min)	65 (61–74)	66 (60.2–75)	65 (62–72.7)	0.56	0.145	0.048
***Comorbidities***						
Coronary artery disease, N (%)	80 (44.4)	33 (35.9)	47 (53,4,6)	0.02	0.358	0.051
Diabetes, N (%)	50 (27.8)	26 (28.3)	24 (27.3)	0.88	0,022	0.093
Dyslipidemia, N (%)	140 (77.8)	69 (75)	71 (80.7)	0.36	0.137	0.068
Smoking habit, N (%)	89 (49.4)	44 (47.8)	45 (51.1)	0.66	0.066	0.070
Hypertension duration (years)	11 (5.2–17.8)	10 (5–15)	14 (8–19.5)	0.07	0.256	0.045
Thyroid disorders, N (%)	30 (16.7)	16 (17.4)	14 (15,9)	0.79	0.040	0.048
***Laboratory data***						
Glycosylated hemoglobin (HbA1c), %	5.8 (5.3–6.3)	5.8 (5.4–6.2)	5,8 (5.1–6.3)	0.68	0.025	0.085
LDL cholesterol, mg/dl	79.5 (51.2–106.5)	85 (51–111.5)	75,5 (52–100.7)	0.12	0.231	0.028
eGFR, ml/min	89 (75–98)	90 (75,2–99,7)	88,5 (72.2–97)	0.27	0.172	0.027
Hemoglobin, g/dl	13.6 ± 1.4	13,7 ± 1,3	13,6 ± 1.5	0.45	0.113	0.043
***Echocardiographic data***						
EF, %	58 (55–60)	60 (55.2–62)	55 (54–59.7)	<0.001	0.848	0.143
LV hypertrophy, N (%)	47 (26.1)	19 (20.7)	28 (31.8)	0.09	0.256	0.045
LAV index, ml/m^2^	30 (26–35)	29.5 (25–35)	30 (26–35)	0.33	0.132	0.001
***Medical therapy***						
Aspirin, N (%)	113 (62.8)	47 (51.1)	66 (75)	0.001	0.511	0.078
P2Y12 inhibitor, N (%)	52 (28.9)	20 (21.7)	32 (36.4)	0.03	0.326	0.005
ACE inhibitors, N (%)	83 (46.1)	46 (50)	37 (42)	0.28	0.160	0.032
Mineralcorticoid receptor antagonist (MRA), N (%)	9 (5)	4 (4.3)	5 (5,7)	0.68	0.061	0.004
β-Blockers, N (%)	100 (55.6)	50 (54.3)	50 (56.8)	0.74	0.050	0.019
Morisky scale	4 (3–4)	4 (4–4)	4 (3–4)	0.01	0.418	0.070
**Polypharmacy categories**						
1–3	41 (22.8)	28 (30.4)	13 (14.8)	0.04	0.391	0.044
4–6	90 (50)	43 (46.7)	47 (53.4)			
>6	49 (27.2)	21 (22.8)	28 (31.8)			

*ACE* angiotensin converting enzyme, *ARBs* angiotensin receptor blockers, *BMI* body mass index, *DBP* diastolic blood pressure, *eGFR* Estimated Glomerular Filtration Rate, *GLS* Global Longitudinal Strain, *HbA1c* Glycosylated hemoglobin, *HR* heart rate, *LAV* left atrial volume, *LDL* low-density lipoprotein, *LV* left ventricle, *MRA* mineralcorticoid receptor antagonist, *SMD* standardized mean difference, *SBP* systolic blood pressure

Covariates in the weighted model were selected based on previous studies on factors associated with GLS and/or the study outcome. The balance between the GLS subgroups was assessed using weighted standardized mean difference (SMD), and graphically represented by density and love plots [25]. For each variable, the absolute standardized mean difference represents the absolute difference between the mean values in the two groups divided by the common SD. An SMD value < 0.1 was considered acceptable. In the weighted comparative samples, we used a logistic regression model to estimate the weighted odds ratio (wOR) and their 95% CI for the study outcome using a robust variance estimator [26]. Residual differences between GLS groups after IPTW were adjusted by forcing the insufficiently balanced variables into the weighted model (providing adjusted wOR) [27]. As a sensitivity analysis, we used several different strategies: (1) we fitted a multivariable logistic regression model to assess the effect of GLS on mild cognitive impairment in the non-weighted population, using covariates that showed a *p* < 0.05 in the univariate analysis and forcing only the LVEF; (2) we employed a nonparametric bootstrapping approach to calculate point estimates and CIs, drawing 1000 random samples with replacement after IPTW [28]; (3) we developed a different IPTW model using, as GLS cut off value, the best threshold to predict MCI, identified by the highest value of the Youden J index in the receiver operating characteristic (ROC) curve analysis [29]; (4) we calculated the E-value, defined as the minimum strength of association on the risk-ratio scale that an unmeasured confounder would need to have with both the treatment assignment and the outcome, to reverse a specific treatment-outcome association, conditional on the measured covariates [30]. Finally, in order to better define the potential incremental value of GLS in comparison to conventional echocardiographic parameters, we analysed different predictive models defined by both clinical characteristics (age and gender) and echocardiographic parameters (EF, diastolic grading, E/e’ ratio, LAVi, LVMi) and tested the discriminatory ability of each model, and its modification by adding the GLS, calculating the difference of the area under the ROC curve (AUC), using the DeLong’s test. Statistical analyses were conducted using SPSS software version 25.0 (SPPS Inc., Chicago, Illinois) and R version 4.0.5 (R Foundation for Statistical Computing, Vienna, Austria, using, specifically, the WeightIt package to create the pseudo population, the Cobalt package to assess the success in achieving covariate balance, and the Evalue package to calculate bounds and E-values for unmeasured confounding). A *p* value of less than 0.05 was considered significant.

## Results

Table 1 presents the baseline demographic and clinical characteristics of the whole study cohort and according to the GLS median value. Among the 180 patients enrolled, almost 41,1% were female, and the mean age was 65,6 ± 7,2. We detected an MCI condition in 39 study participants (21.7%).

The study selection process is shown in Supplementary Fig. 1. As part of the study selection process, 9 patients were excluded from the overall study population due to inadequate echocardiographic images to perform GLS estimation.

When the study population was stratified according to the GLS median value, significant differences were detected in age, gender, prevalence of ischemic heart disease, ejection fraction, and medical treatment (Table 1). We also detected an almost significant difference in the duration of hypertension history and prevalence of left ventricle hypertrophy (Table 1).

Extensive analysis of echocardiographic parameters in the whole study cohort and according to the GLS median value is presented in the supplementary Table 1. The median GLS value was 18% (IQR: 16,5–19,7), and the median LVEF was 58% (IQR: 55–60).

**Fig. 1:**
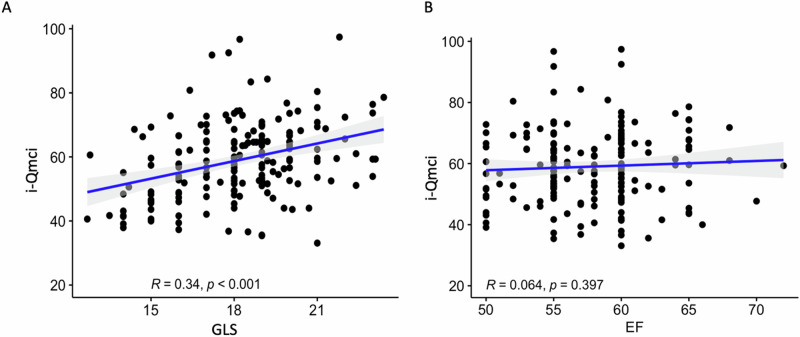
Representation of the correlation, with the relative coefficients, between the GLS* (**A**) and the EF (**B**) with the i-Qmci. *We used the GLS absolute value for easier interpretation. GLS global longitudinal strain, EF ejection fraction, i-Qmci Italian version of quick mild cognitive impairment

The outcome analysis showed a higher prevalence of MCI in patients with lower GLS values (Table 2), and the GLS was significantly correlated with the Qmci-I score, as clearly outlined in Fig. 1A. On the contrary, the LVEF did not show a significant correlation with the Qmci-I (Fig. 1B).

**Table 2:** Outcome analysis

Variables	Whole study population (*N* = 180)	GLS > 18% (*N* = 92)	GLS ≤ 18% (*N* = 88)	*P* value
Quick mild cognitive impairment (Qmci-I) score^a^	59 ± 12	62,1 ± 11,7	55,7 ± 11,6	<0.001
Mild cognitive impairment (MCI)^b^	39 (21.7)	10 (10.9)	29 (33)	<0.001

*GLS* global longitudinal strain, *MCI* mild cognitive impairment, *Qmci-I* Italian version of quick mild cognitive impairment

^a^Corrected by age and education level

^b^MCI condition was defined by a total Qmci corrected for age and education <49.4

**Fig. 2:**
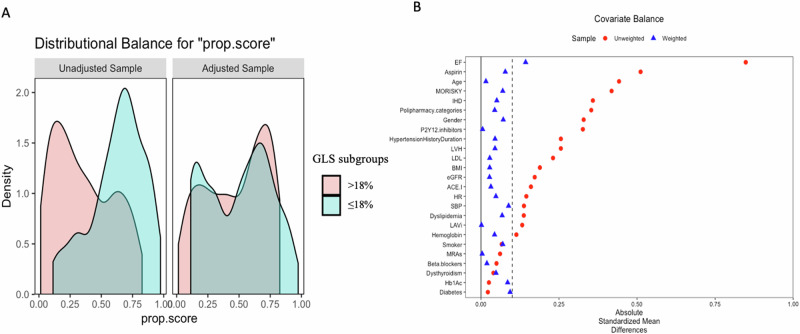
**A** Propensity score distributional overlap before (unadjusted) and after (adjusted) propensity score weighting. **B** Love plot for standardized mean differences comparing covariate values before (red circle) and after (blue triangle) propensity score weighting. A standardized mean differences <0.1 was considered acceptable to support the assumption of balance between the GLS sub-groups

Regarding the IPTW population, the absolute standardized difference showed that the populations were generally well-balanced in both groups of patients with GLS > 18% and GLS ≤ 18%, except for the value of ejection fraction (Table 1 and Fig. 2A, B).

### Outcome analysis after IPTW and sensitivity analysis

The GLS was significantly associated with the study endpoint (OR, 1,22 [95% CI, 1.07–1.39], *P* = 0.003; Table 3). Further adjustment for the residual differences (after IPWT) did not alter the results (adjusted wOR, 1.23 [95% CI, 1.07–1.40], *P* = 0.003; Table 3).

**Table 3:** Logistic regression analysis: odds ratios for MCI in patients with GLS ≤ 18% vs. patients with GLS > 18%

Analysis	OR (95% CI)	*P* value	Bootstrap adjusted OR (bootstrap 95% CI)^a^
IPTW	1.22 (1.07–1.39)	0.003	1.21 (1.01–1.56)
adjusted IPTW^b^	1.23 (1.07–1.40)	0.003	1.22 (1.01–1.53)

*CI* confidence intervals, *IPTW* inverse probability of treatment weighting, *GLS* global longitudinal strain, *MCI* mild cognitive impairment, *OR* odd ratio

^a^Bootstrap adjusted ORs and 95% CIs were estimated using 1000 bootstrap samples

^b^adjusted for EF, which was not perfectly balanced after IPTW

The sensitivity analyses confirmed the results found in the propensity weighting model (Tables 3, 4). The univariate logistic regression analysis (Supplementary Table 2) identified the following predictors: age, heart rate, Morisky scale, and the GLS. When we performed the multivariate logistic regression model forcing also the LVEF, the GLS ≤ 18% was still significantly associated with the outcome of interest, with an OR of 4.29 (95% CI: 1.69–10.9; *P* = 0.002, Table 4). The bootstrapping also confirmed the robustness of the IPTW results, showing small change in the point estimate and confidence intervals (Table 3). After identification of the best GLS threshold in predicting MCI (supplementary Fig. 2), we performed a further sensitivity analysis using this new cut-off in order to create new propensity weighted sample. When we applied a logistic regression model in this new weighted sample, the results were still consistent (Table 4). The calculated E-value was 1,74, indicating that to undermine the estimate, there should be unmeasured confounders linked to both the outcome and the exposure at a minimum of 1,74 times the measured confounders, with an OR of 1.2 after the IPTW analysis. Finally, in order to better define the incremental value of the GLS in comparison to traditional echocardiographic parameters, we analysed the modification in the discriminatory ability of a predicting model, including age, gender, and conventional echocardiographic parameters after the addition of the GLS (Supplementary Fig. 3). The analysis showed a significant incremental value of the GLS (Supplementary Fig. 3).

**Table 4:** Sensitivity analysis: odds ratios for MCI in patients with GLS ≤ 18% vs. patients with GLS > 18%, and in patients with GLS ≤ 16.1% after propensity weighting

Analysis	OR (95% CI)	*P* value
Unweighted univariate logistic regression	4.03 (1.82–8.91)	<0.001
Unweighted multivariate logistic regression^a^	4.29 (1.69–10.9)	0.002
IPTW with GLS cut off of 16,1%	1.62 (1.28–2.06)	0.001

*CI* confidence intervals, *GLS* global longitudinal strain, *MCI* mild cognitive impairment, *OR* odd ratios

^a^Adjusted for the following variables: Age, Heart rate, Morisky scale, (which showed a *P* < 0.05 at the univariate analysis), and EF

## Discussion

Two main findings emerge from our analysis: (1) 22% of hypertensive patients show signs of MCI, according to the Qmci-I; (2) subclinical LV dysfunction, detected by the GLS, is associated with MCI.

Regarding the first point, the prevalence of cognitive impairment in hypertensive patients is highly variable in literature due to differences in population characteristics and in tools utilized to assess cognitive functions among studies [31]. Nevertheless, our results align with previous studies [32, 33], where the prevalence of cognitive impairment in hypertensive patients is around 20–30%.

Our study focused on finding a parameter associated with MCI rather than with overt dementia, since this approach is crucial in clinical practice to establish early cognitive dysfunction in order to identify hypertensive patients with a higher risk of progressive brain functional impairment.

Concerning the role of subclinical LV dysfunction, measured by the GLS, in the prediction of MCI and silent cerebrovascular disease, to the best of our knowledge, only a couple of studies have investigated this aspect [16, 34]. Both studies suffer from an essential bias due to selecting a relatively healthy sample with a lower cardiovascular risk profile than the general real-world population. Indeed, the prevalence of coronary artery disease was only 3% for the first study, while it was among the exclusion criteria in the second one. Our study showed that GLS is independently associated with MCI despite a not negligible burden of cardiovascular comorbidity, with 44,4% of patients affected by coronary artery disease. Moreover, we used a propensity weighting analysis to correct for the several confounders measured in our study cohort. Therefore, we can affirm that our results strengthen the value of GLS as a valuable tool for assessing pre-clinical cognitive and cardiac dysfunction. Another important finding of our analysis is that only GLS was associated with MCI, not the LVEF. The LVEF has been associated with cognitive impairment in previous reports [35, 36]. Still, it represents a mid-to-late feature of impaired myocardial contractility [37] with limited predictive power, especially if the goal is to detect cognitive dysfunction in its early stage.

Moreover, the LVEF is load-dependent, influenced by heart rate, and may not accurately reflect cardiac contractility, especially in cardiac hypertrophy [38]. On the contrary, the GLS has been widely recognized as a more effective technique than conventional LVEF in detecting subtle changes in LV function in the context of several cardiovascular diseases [39] due to its property of detecting abnormalities of the longitudinal myocardial fibers located predominantly in the sub-endocardium [38], which represents the wall layer most susceptible to ischemia [40]. The exact mechanisms accounting for the associations between subclinical cardiac dysfunction and cognitive impairment are generally considered multifactorial but remain elusive. Previous studies have observed an association between GLS impairment and unfavorable cerebral structural and hemodynamic changes [34, 41, 42].

Russo and colleagues reported that a lower GLS is associated, through direct and indirect mechanisms, with subclinical brain diseases, including silent infarcts and white matter volume hyperintensity, consequently affecting brain health [34].

An alternative explanation could be related to hemodynamic consequences of subclinical LV dysfunction, such as a decreased stroke volume, that may influence the autoregulatory mechanisms to preserve cerebral blood flow and directly impact brain tissue [41, 43]. Indeed, brain regions with a less extensive network of collateral sources of blood flow, including the temporal lobes, appear particularly vulnerable to compromised cognitive performance [41, 42].

Moreover, these unfavorable structural and hemodynamic changes may affect the synthesis of brain proteins required for synaptic plasticity, potentially detrimentally affecting cognitive functions [5, 9].

These pathophysiological mechanisms could explain the close relationship between subclinical left ventricular dysfunction defined by GLS and MCI in hypertensive patients.

In conclusion, our results highlight that the GLS is a potential predictor of MCI and, therefore, a valuable tool for establishing preventive strategies to arrest the progression toward a cognitive decline in hypertensive patients.

### Strengths

Our study has several strengths. First, we recruited hypertensive patients without clinical dementia, stroke, medical diseases, and/or drugs with known significant effects on cognition but with a not negligible comorbidity burden to avoid a possible “hyper-normality bias”. Second, GLS provides a more reliable measure of subclinical cardiac dysfunction than LVEF. Furthermore, we used a propensity weighting analysis to correct for possible confounding and several statistical approaches to confirm the robustness of our primary analysis, such as a traditional multivariable logistic regression model and a bootstrapping approach due to the relatively small sample size. We also assessed the potential value that a hypothetical unmeasured factor should have to nullify our results, finding that value equal to 1.74, which is a not negligible order factor if compared with the ORs detected in our analysis. An additional strength includes utilizing a comprehensive, rapid, multi-domain cognitive screening instrument adjusted for age and education.

### Limitations

Our results should be interpreted considering some limitations. First, this study involves a relatively small sample size. However, this study applies rigorous exclusion criteria with known significant effects on cognition to reduce confounding factors that complicate the recruitment of a large population of hypertensive patients. Therefore, the generalizability of the results could be improved. Second, the cross-sectional design precludes causal assumptions, and longitudinal and/or prospective trial data will be necessary to clarify a causality relationship between the observations detected in our analysis.

Also, our study does not provide a centralised analysis of echocardiographic data in a core laboratory.

Another limitation concerns the absence of brain magnetic resonance imaging information, which was not performed in our study. However, our study was not conceived to evaluate the association of the GLS with brain magnetic resonance imaging information but to investigate the role of the GLS in predicting an MCI, as defined by the Qmci-I. Finally, we do not have information on the atherosclerotic burden in our hypertensive population due to missing information about ankle/brachial index and carotid ultrasonography, which have been associated with subclinical atherosclerotic disease, and we do not provide information about the microvascular function which could be interesting in the effort to find a pathogenetic link between the GLS and the brain function. Indeed, we cannot rule out that the GLS could be an expression of a more extensive microvascular dysfunction potentially affecting different organ functions, including the brain; however, a deep investigation of microcirculation in various organs was prohibitive in our case, and it will require a different study design.

## Conclusion

In a real-world study, including hypertensive patients without medical diseases and/or drugs with known significant effects on cognition but with a not negligible comorbidity burden, we demonstrated a strong significant association between subclinical cardiac dysfunction quantified by GLS and the MCI.

Our study suggests that GLS is an additional parameter of utmost importance in clinical practice for early recognition of MCI and should be carefully and systematically assessed in these peculiar hypertensive patients. However, studies on a larger population will be needed to confirm this association.

### Perspective

In this real-world study, including hypertensive patients without medical diseases and/or drugs with known significant effects on cognition but with a not negligible comorbidity burden, subclinical cardiac dysfunction defined by GLS is strongly correlated with MCI, representing, therefore, a valuable tool for establishing preventive strategies to arrest the progression toward a cognitive decline.

## Supplementary information

Supplemental Material

Supplementary figure 1

Supplementary figure 2

Supplementary figure 3
