# Supplementary material for: Subclinical left ventricular dysfunction assessed by global longitudinal strain correlates with mild cognitive impairment in hypertensive patients
Source: Hypertens Res. 2025 Mar 17;48(5):1768–78. doi: 10.1038/s41440-025-02182-3 (PMC12055581; doi:10.1038/s41440-025-02182-3)
Supplement: Supplementary file 1 — Supplemental Material [file 41440_2025_2182_MOESM1_ESM.docx]

**SUPPLEMENTAL MATERIAL**

**Subclinical left ventricular dysfunction assessed by global longitudinal strain correlates with mild cognitive impairment in hypertensive patients**

**TABLE OF CONTENTS**

Supplementary Table 1. Echocardiographic parameters in the whole study cohort, and according to GLS ≤ and > 18%

Supplementary Table 2. Univariate logistic regression analysis for the prediction of MCI by specific baseline demographic, clinical, laboratory and echocardiographic data.

Supplementary figure legends

**Supplementary** **Table 1**

Echocardiographic parameters in the whole study cohort, and according to GLS ≤ and > 18%

| **Variables** | **Whole study population (N=225)** | **GLS > 18% (N=92)** | **GLS ≤ 18% (N=88)** | **P-value** |
| --- | --- | --- | --- | --- |
| Ejection fraction (%) | 58 (55-60) | 60 (55,2-62) | 55 (54-59,8) | <0,001 |
| LV GLS (%)* | 18 (16,5, 19,7) | 19,6 (19-20,9) | 16,4 (15-17,9) | <0,001 |
| Stroke volume, ml | 76 (65,2-81) | 76 (67-81) | 74,5 (65-79) | 0,22 |
| LVDd, mm | 45 (41-48) | 44 (41-47,8) | 45 (41,2-49) | 0,40 |
| IVSd, mm | 11 (10-13) | 11 (10-12) | 12 (10,2-13) | 0,002 |
| LVPWd, mm | 10 (9-12) | 10 (8-11) | 11 (9-13) | 0,001 |
| LVMI | 92,6 ± 25,5 | 86,8 ± 24,8 | 98,7 ± 24,8 | 0,002 |
| LV hypertrophy, N (%) | 47 (26,1) | 19 (20,7) | 28 (31,8) | 0,09 |
| Concentric hypertrophy, N (%) | 37 (20,6) | 16 (17,4) | 21 (23,9) | 0,28 |
| Eccentric hypertrophy, N (%) | 10 (5,6) | 3 (3,3) | 7 (8) | 0,17 |
| LVEDV index, ml/m^2^ | 52 (45,2-59,8) | 49 (45-58,8) | 54 (47-60) | 0,08 |
| LAV index, ml/m^2^ | 30 (26-35) | 29,5 (25-35) | 30 (26-35) | 0,33 |
| E/A | 0,82 (0,7-1,03) | 0,84 (0,71-1,08) | 0,8 (0,66-1,02) | 0,15 |
| DT, msec | 210,5 (185,5-243) | 210,5 (180,2-238,8) | 210,5 (189,2-250) | 0,38 |
| E/e’ | 8 (6,7-10) | 8 (6,7-10) | 8 (6,6-10) | 0,24 |
| Diastolic dysfunction | | | | |
| Type I | 69 (38,3) | 43 (46,7) | 26 (29,5) | 0,12 |
| Type II | 9 (5) | 4 (4,3) | 5 (5,7) |  |
| Type III | 3 (1,7) | 1 (1,1) | 2 (2,3) |  |
| TAPSE (mm) | 22 (20-24) | 22,5 (20-24) | 22 (21-24) | 0,30 |
| RVs’ (cm/sec) | 12 (11-13) | 12 (11-13) | 12 (11-13) | 0,74 |
| PAPs (mmHg) | 28 (25-32) | 27 (24-32) | 30 (25-34,2) | 0,03 |

DT: deceleration time; IVSd: Interventricular septal end-diastole; LVDd: left ventricular diameter end-diastole; LVEDV: left ventricular end-diastolic volume; GLS: global longitudinal strain; LVEF: left ventricular ejection fraction; LVMI: left ventricular mass index; LVPWd: left ventricular posterior wall end-diastole; LV: left ventricular; LAV: left atrial volume. TAPSE: Tricuspid Annular Plane Systolic Excursion; PAPs: Pulmonary artery systolic pressure.

*We used the GLS absolute value for easier interpretation.

**Supplementary Table 2**

Univariate logistic regression analysis for the prediction of MCI by specific baseline demographic, clinical, laboratory and echocardiographic data.

| Variables | OR (95% CI) | P-value |
| --- | --- | --- |
| ***Demographic and clinical data*** |  |  |
| Age | 1,07 (1,017-1,127) | 0,009 |
| Female gender | 0,87 (0,42-1,797) | 0,70 |
| Body mass index (BMI) | 1,025 (0,939-1,119) | 0,58 |
| Systolic blood pressure (mmHg) | 0,990 (0,968-1,012) | 0,38 |
| Diastolic blood pressure (mmHg) | 0,971 (0,934-1,009) | 0,13 |
| Heart rate (beats per minute) | 0,951 (0,912-0,992) | 0,02 |
| ***Comorbidities*** |  |  |
| Coronary artery disease | 0,957 (0,468-1,955) | 0,90 |
| Diabetes | 1,027 (0,467-2,262) | 0,95 |
| Dyslipidemia | 1,397 (0,565-3,456) | 0,47 |
| Smoking habit | 0,741 (0,363-1,512) | 0,41 |
| Hypertension duration (years) | 1,015 (0,967-1,066) | 0,55 |
| Thyroid disorders | 0,89 (0,33-2,35) | 0,81 |
| ***Laboratory data*** |  |  |
| Glycemia, mg/dl | 1,002 (0,986-1,018) | 0,81 |
| Glycosylated hemoglobin (HbA1c), % | 0,790 (0,435-1,435) | 0,44 |
| LDL cholesterol, mg/dl | 0,999 (0,989-1,009) | 0,78 |
| eGFR, ml/min | 1,011 (0,992-1,030) | 0,27 |
| Haemoglobin, g/dl | 0,851 (0,665-1,087) | 0,19 |
| ***Echocardiographic data*** |  |  |
| Ejection fraction (%) | 1,005 (0,928-1,089) | 0,90 |
| LV GLS < 18% | 4,03 (1,824-8,906) | 0,001 |
| LV hypertrophy | 1,346 (0,617-2,936) | 0,45 |
| LAV index, ml/m^2^ | 0,991 (0,945-1,040) | 0,72 |
| Diastolic dysfunction | 0,806 (0,461-1,411) | 0,45 |
| ***Medical therapy*** |  |  |
| Aspirin | 1,677 (0,773-3,639) | 0,19 |
| P2Y12 inhibitor | 1,518 (0,715-3,222) | 0,28 |
| Statin | 1,397 (0,565-3,456) | 0,47 |
| ACE inhibitors | 1,487 (0,729-3,030) | 0,27 |
| Angiotensin receptor blockers (ARBs) | 0,850 (0,407-1,775) | 0,66 |
| Mineralcorticoid receptor antagonist (MRA) | 0,438 (0,053-3,608) | 0,44 |
| β-Blockers | 0,916 (0,449-1,866) | 0,81 |
| Insulin | 1,385 (0,350-5,491) | 0,64 |
| Calcium Channel Blockers (CCBs) | 1,39 (0,67-2,9) | 0,37 |
| Diuretics (Thiazide) | 1,04 (0,43-2,5) | 0,93 |
| Morisky scale | 0,516 (0,323-0,825) | 0,006 |
| Polypharmacy categories | 1,421 (0,851-2,372) | 0,18 |

ACE, Angiotensin Converting Enzyme; ARBs, Angiotensin receptor blockers; BMI, Body mass index; DBP, Diastolic Blood Pressure; eGFR, Estimated Glomerular Filtration Rate; GLS, global longitudinal strain; HbA1c: Glycosylated hemoglobin; HR, Heart Rate; LAV, Left Atrial Volume; LDL, Low-density Lipoprotein; LV, Left Ventricle; MRA, Mineralcorticoid receptor antagonist; OR, odd ratio; SBP, Systolic Blood Pressure.

**SUPPLEMENTARY FIGURE LEGENDS**

**Supplementary Fig. 1: Study selection process**

**GLS, global longitudinal strain**

**Supplementary Fig. 2: Receiver operating characteristics (ROC) curves analysis for GLS to predict the occurrence of mild cognitive impairment (MCI)**

**GLS, global longitudinal strain**

**Supplementary Fig. 3: Receiver operating characteristics (ROC) curves analysis according to different models**

**GLS, global longitudinal strain; EF, ejection fraction;** **LVMI: left ventricular mass index; LAVi, left atrial volume index**
