# Supplementary figures and images for: Subclinical left ventricular dysfunction assessed by global longitudinal strain correlates with mild cognitive impairment in hypertensive patients
Source: Hypertens Res. 2025 Mar 17;48(5):1768–78. doi: 10.1038/s41440-025-02182-3 (PMC12055581; doi:10.1038/s41440-025-02182-3)

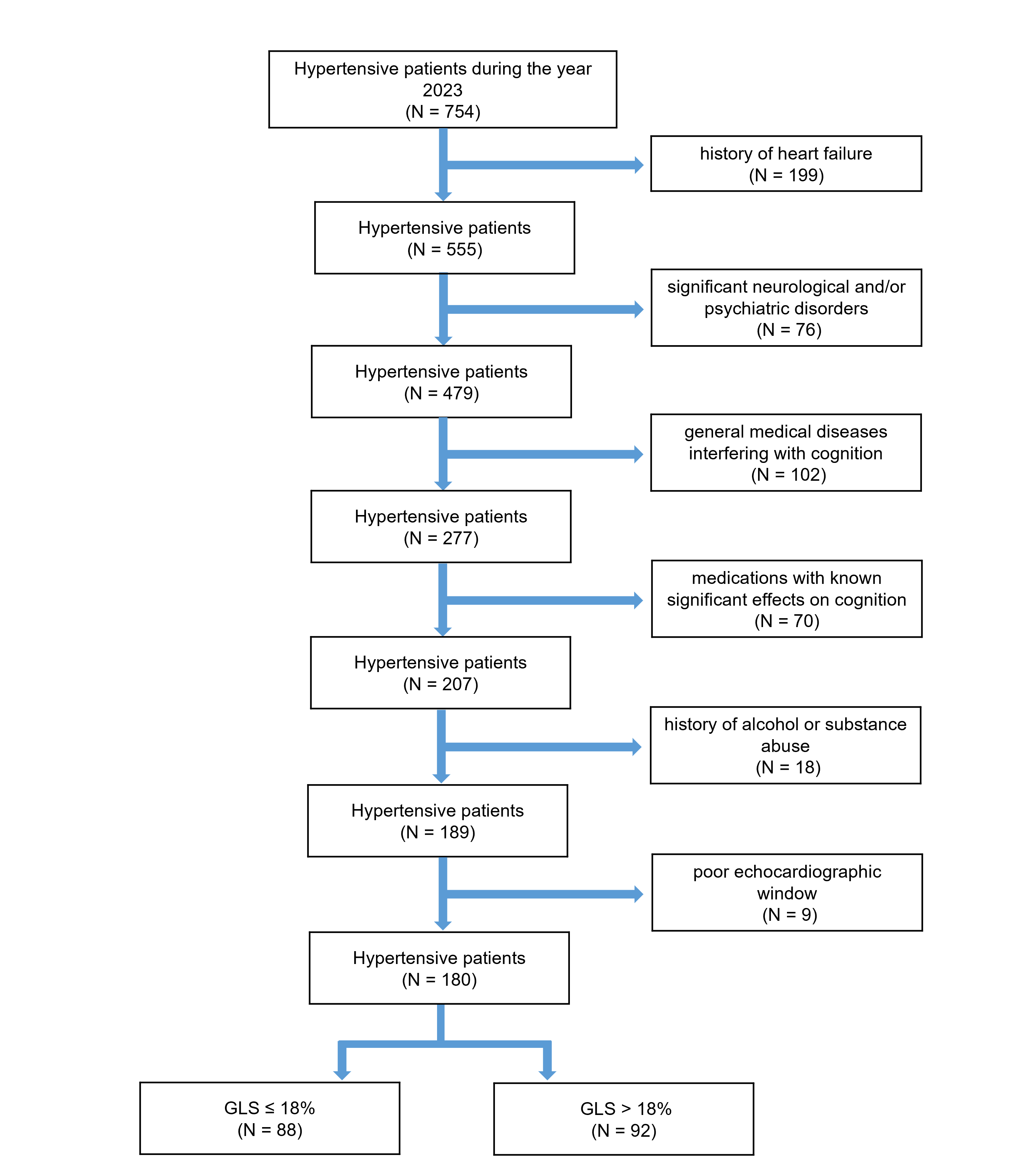

Supplement: Supplementary file 2 — Supplementary figure 1 [file 41440_2025_2182_MOESM2_ESM.tif]

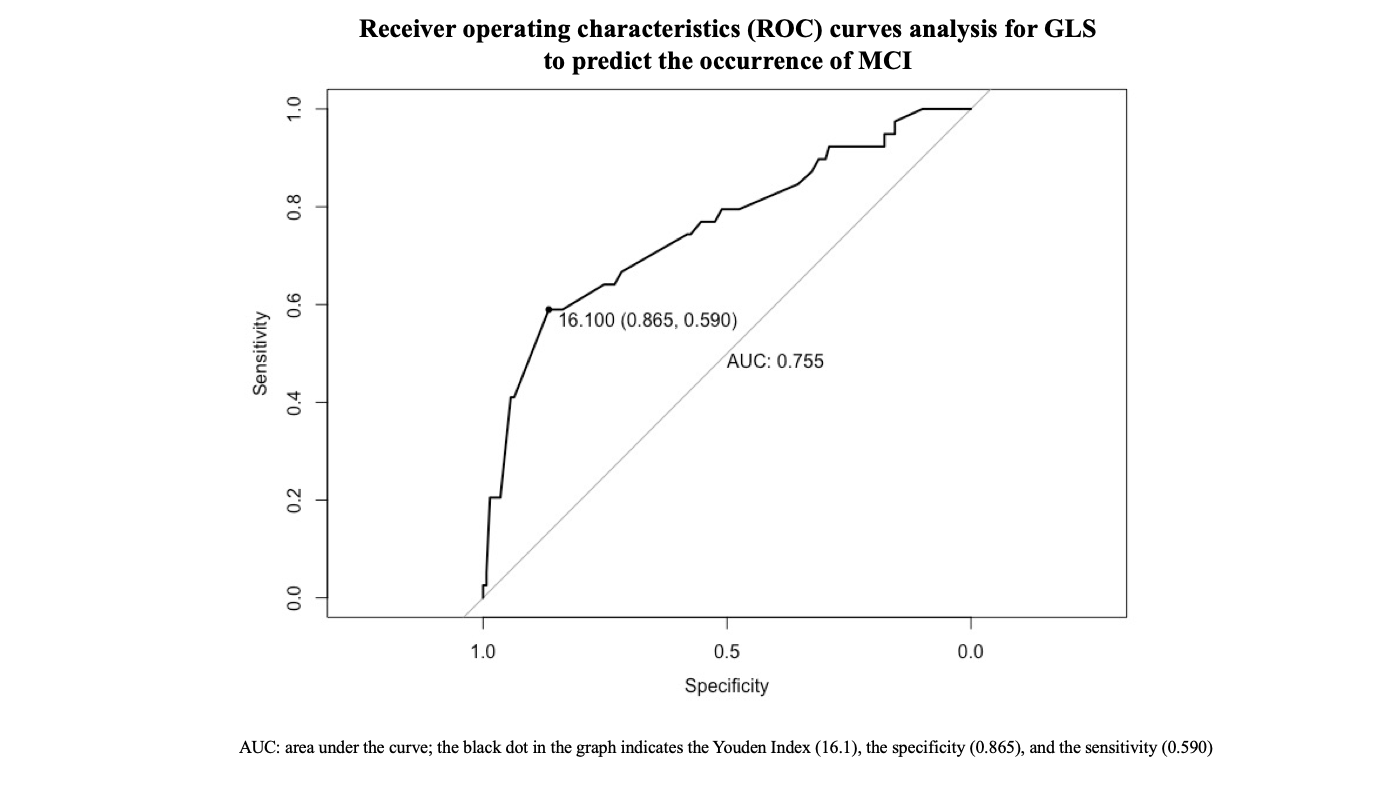

Supplement: Supplementary file 3 — Supplementary figure 2 [file 41440_2025_2182_MOESM3_ESM.tiff]

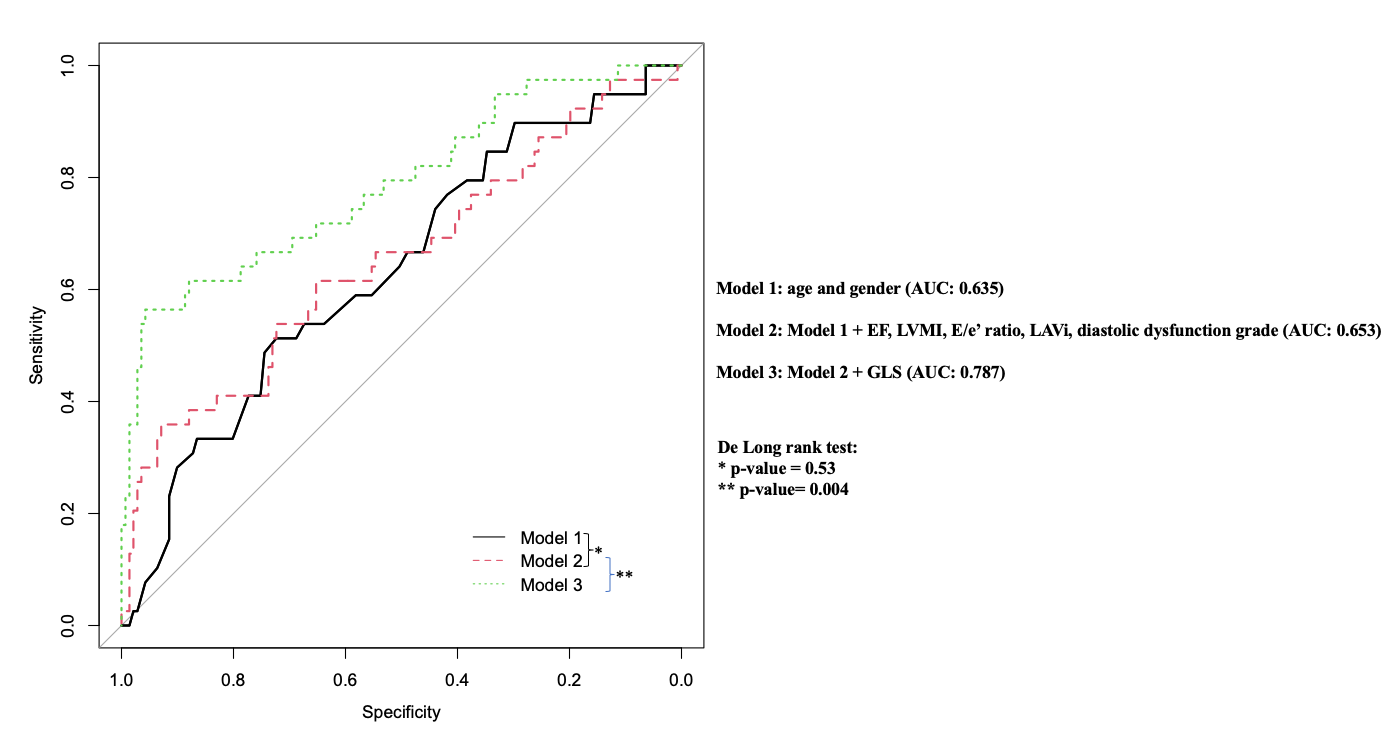

Supplement: Supplementary file 4 — Supplementary figure 3 [file 41440_2025_2182_MOESM4_ESM.tiff]
